# Back pain and radicular pain after lumbar microdiscectomy

**DOI:** 10.1186/s12893-023-02114-3

**Published:** 2023-07-26

**Authors:** Konsta Koivunen, Katri I. Pernaa, Mikhail Saltychev

**Affiliations:** 1 Clinical Division, University of Turku, Turku, Finland; 2 Department of Orthopedics, Turku University Hospital and University of Turku, Turku, Finland; 3 Department of Physical and Rehabilitation Medicine, Turku University Hospital and University of Turku, PO Box 528, Turku, FI-20701 Finland

**Keywords:** Diskectomy [MeSH], Microsurgery [MeSH], Low Back Pain [MeSH], Musculoskeletal Pain [MeSH], Pain Management [MeSH]

## Abstract

**Purpose:**

It is generally expected that lumbar microdiscectomy affects radicular leg pain, but not so much local back pain. The primary objective was to evaluate if the trajectories of changes in pain severity follow similar patterns for back and radicular leg pain after lumbar microdiscectomy. The secondary objective was to investigate the associations between some preoperative parameters and the patterns of these trajectories.

**Methods:**

Register-based retrospective study of 353 patients undergoing microdiscectomy in the lumbar spine. Linear mixed modelling was applied.

**Results:**

The average age of the participants was 46 years and 44% were women. The developmental trajectories were similar for both back and leg pain. Pain level decrease during the first year after the surgery, slightly worsening later. No statistically significant interactions were detected of preoperative pain duration or severity, sex or age on the shapes of the trajectories. For every analyzed grouping factor, the 95% confidence intervals overlapped at every postoperative time point with one exception – worse preoperative back pain was statistically significantly associated with worse pain at three months and at the end of the two-year follow-up.

**Conclusion:**

After microsurgical discectomy, developmental curves for both back and radicular leg pain demonstrated similar patterns. Pain intensity decreased during the first year after the surgery. and slightly increased after that remaining, however, below the preoperative level. Age, sex, preoperative pain duration or preoperative intensity of leg pain were not associated with significant differences in the trajectories of pain severity after the surgery. In this study, severe preoperative back pain was the only factor, which was significantly associated with worse postoperative trajectory of pain intensity.

## Introduction

Microsurgical excision of herniated lumbar intervertebral disc is a widely accepted generally safe and often effective treatment to ease back and leg pain intensity and disability severity [1–7]. However, while multiple studies have reported significant improvement after the procedure concerning both the severity of pain and the level of disability, it has usually been reported that the procedure might affect radicular leg pain more substantially than local back pain. Osterman et al., Peul et al. and Hareni et al. have reported that in a one-year follow-up after microdiscectomy, leg pain decreased down to 10%-30% of the initial level, while the change in back pain intensity was more modest – pain remained at 30%-60% of the initial level [2, 3, 8]. Other studies have suggested that a change in severity of back pain might be similar comparing to a change in leg pain intensity [9, 10]. In a one-year follow-up after discectomy, Iorio-Morin et al. have observed similar trajectories for both leg pain and back pain [9]. Respectively, Toyone et al. have introduced a potentially equal effect of microdiscectomy on both back and leg pain: in a 40-month follow-up, leg pain decreased from 87/100 to 8/100 points, while the respective decrease in back pain has been from 51/100 to 10/100 points [10]. Thus, there has been uncertainty if discectomy has equal effects on leg and back pain.

Age might be a significant factor affecting the magnitude of postoperative pain decline. Changes in both pain and disability have been found to be greater in children and adolescents compared to adults [6, 11, 12]. The corresponding role of sex has been studied only a little and mostly on small samples [13, 14]. One study has reported that women might use more pain medications as well as have worse back and leg pain and poorer functioning and quality of life one year after lumbar disc herniation surgery [15]. Otherwise, no differences between sexes in pain development after surgery has usually been noticed [13, 14]. Surgical intervention is usually considered after at least six weeks after the onset of pain symptoms [1]. Limited evidence has linked lengthy preoperative pain with poorer outcomes of surgery [8, 16, 17].

Establishing connection between preoperative status and outcome may help to select those groups, which have a greater potential to benefit from surgery, and, in turn, to avoid, when plausible, groups with improbable improvement. Additionally, familiarity with the trajectories of pain and disability in postoperative development may help to allocate rehabilitation measures with more precision. Also, this knowledge may be of help when planning a schedule for follow-up measures. The primary objective was to evaluate if the trajectories of changes in pain severity follow similar patterns for back and radicular leg pain after lumbar microdiscectomy. The secondary objective was to investigate the associations between some preoperative parameters and the patterns of these trajectories.

## Methods

The data were obtained from a register containing data on patients undergoing spinal surgery of any kind between June 21, 2018 and August 17, 2021 at the Turku University Hospital, Finland. The patients responded to repeated surveys a) <  = 2 months before surgery (timepoint #0); 2 to 4 months after surgery (timepoint #1); 11 to 13 months after surgery (timepoint #2); and 23 to 25 months after surgery (timepoint #3). The survey contained questions on demographics and the severity of disability and pain. In this study, a patient was included if the procedure code was “ABC16 Microsurgical excision of lumbar intervertebral disc displacement”, according to the Nordic Classification of Surgical Procedures (NCSP), version 1.15. The indications for surgery have varied. While the register does not cover the exact details on the reasons for surgical decision, there are three main indications for discectomy in Finland: 1) cauda equina syndrome, 2) progressive motor deficiency in lower extremities and 3) persistent pain. First two indications are relatively rare. Thus, with great certainty, it could be assumed that most of the patients have gone through discectomy due to major persistence of pain. By the decision of the institutional ethical board, register-based studies in the institution do not require a separate statement of approvement. All data used for the analysis were extracted and processed anonymously.

Age was defined in full years at the time of surgery. Body mass index (BMI) was defined as body weight divided by a squared height and expressed in kg/m^2^. The duration of pain preceding the time of surgery was defined as < 6 weeks, 6–12 weeks, 3–12 months, or > 1 year. The duration of pain was further dichotomized as <  = 1 year vs. > 1 year. Pain intensity was assessed by using a visual analogue scale from 0 to 100 points with 0 indicating no pain and 100 indicating most possible pain. To ease the interpretation of the results when exploring the associations between preoperative characteristics and pain trajectories, age, preoperative severity of back and leg pain were dichotomized based on their means. The severity of disability was assessed by using the composite score of the Oswestry Disability Index (ODI) with a score of 0% representing the highest possible level of functioning and independence while a score of 100% represented the lowest level of functioning and total dependence.

### Statistical analysis

The descriptive characteristics of the sample were reported as absolute number and percentage or as means and standard deviations along with 95% confidence intervals, when appropriate.

Linear mixed models are models containing both fixed effects and random effects [18]. They are a generalization of linear regression allowing the inclusion of random deviations (effects) other than those associated with the overall error term. The method logic is based on the fact that each patient demonstrates some linear trend in the change of pain severity score and that overall score measurements vary from patient to patient. As any regression line, this trend can be described by three parameters – an intercept (baseline level), a slope (the steepness of the regression curve) and a measurement error. A conventional way to assess the measurement error is to compare each individual slope to an average one. This approach does not take into account that the starting point (intercept = baseline level) may be very different for different individuals. If there is a substantial variety between intercepts, then the variance of the estimate may be overestimated leading to wide confidence intervals and to a statistically insignificant result, even if the real change would be significant if the intercepts were taken into account. A slope describes a so-called “fixed effect”, while an intercept describes a “random effect”. The mixed approach noticed both effects.

The sample was treated as a random sample from a larger population. The model treated the between-patient variability as a random effect (a random-intercept at the patient level). First, two models were compared using a likelihood-ratio test – one model, which assumed that the differences were related to both an intercept and a slope, and another model, which assumed that the differences were related to an intercept (the baseline level) only. The likelihood-ratio test was interpreted based on a two-tailed *p*-value – significant *p*-value < 0.05 would suggest that both an intercept and a slope should be included into a model. However, for both pain variables (back and leg pain), the likelihood-ratio test showed insignificant *p*-values: ~ 1.0 for back pain and 0.16 for leg pain. Thus, only models based on intercepts were included for further analyses.

The MIXED approach was applied in the following steps:

1. The trajectories of pain change over time were created using predicted estimates (margins) along with their 95% confidence intervals (95% CIs) at each time point for the entire sample and for each grouping variable.
2. Differences between the shapes of trajectories were tested by exploring the significance of interaction for each grouping variable.
3. Differences between pain levels at each time point for the entire sample and for each grouping variable were evaluated by examining overlapping 95% CIs.

All the data analyses were performed utilizing Stata 17 (College Station, Texas, U.S.).

## Results

Of the 353 patients, 157 (44%) were women (Table 1). The average age was 46.0 (SD 15.8) years (Table 2). Of the patients, 53% had “M51 Intervertebral disc disorders” and 44% had “G55 Nerve root compression” as main reasons for surgery. About 20% of the patients experienced pain for over one year. The average pain severity (VAS) at the baseline was 59.1 (SD 28.4) for back pain and 68.4 (SD 26.6) points for leg pain. Respectively, the average baseline severity of disability was 47.1 (SD 17.3) points (ODI) referring to moderate or difficult disability. Most of the patients were slightly overweight with average body mass index of 27.7 (SD 5.2) kg/m^2^. There were no significant interactions of sex, age or preoperative pain duration on the baseline severity of back pain with all the *p*-values > 0.05 (Table 3).

**Table 1:** Descriptive characteristics of sample (categorical variables)

Variable	N	%
Gender		
Men	196	56%
Women	157	44%
Total	353	100%
Diagnosis		
M51 Intervertebral disc disorders	188	53%
G55 Nerve root compressions	157	44%
M47 Spondylosis	4	1%
M48 Spondylopathies	2	1%
G83 Paralytic syndromes	1	< 1%
M54 Dorsalgia	1	< 1%
Total	353	100%
Preoperative pain duration		
< = 1 year	277	80%
> 1 year	70	20%
Total	347	100%

**Table 2:** Descriptive characteristics of sample (continuous variables)

Variable	Mean	SD	N
Age group, years			
Age group 1	32.9	6.5	176
Age group 2	59.0	10.9	177
Total	46.0	15.8	353
Body mass index, kg/m^2^	27.7	5.2	353
Oswestry Disability Index, points			
Preoperative	47.1	17.3	320
3 months after surgery	15.3	15.7	182
1 year after surgery	15.3	16.3	132
2 years after surgery	14.4	11.3	43
Back pain			
Preoperative	59.1	28.4	300
Back pain group 1	36.2	21.4	150
Back pain group 2	82.0	10.1	150
3 months after surgery	24.4	25.2	181
1 year after surgery	26.0	26.0	137
2 years after surgery	30.3	26.2	45
Leg pain			
Preoperative	68.4	26.6	298
Leg pain group 1	48.1	23.0	149
Leg pain group 2	88.8	7.6	149
3 months after surgery	26.7	28.2	172
1 year after surgery	24.2	26.6	129
2 years after surgery	28.6	28.0	45

**Table 3:** Pain severity – predicted means and 95% confidence intervals (95% CI)

	Mean	95% CI			Mean	95% CI	
**Back pain**				**Leg pain**			
Entire sample				Entire sample			
Preoperative	58.17	55.15	61.19	Preoperative	67.74	64.68	70.80
3 months	28.18	24.93	31.43	3 months	30.68	27.36	34.00
1 year	21.61	18.20	25.02	1 year	19.37	15.90	22.85
2 years	38.46	31.50	45.41	2 years	33.82	26.54	41.11
Sex				Sex			
Men				Men			
Preoperative	56.30	52.11	60.50	Preoperative	67.15	63.08	71.21
3 months	27.83	23.23	32.43	3 months	30.65	26.14	35.16
1 year	21.14	16.25	26.04	1 year	18.79	14.00	23.59
2 years	36.23	26.27	46.20	2 years	31.58	21.36	41.79
Women				Women			
Preoperative	60.68	56.41	64.95	Preoperative	68.54	63.89	73.19
3 months	28.68	24.18	33.17	3 months	30.78	25.88	35.68
1 year	22.11	17.48	26.74	1 year	20.04	15.01	25.06
2 years	40.98	31.50	50.47	2 years	36.31	25.95	46.67
Age				Age			
Younger				Younger			
Preoperative	60.57	56.37	64.77	Preoperative	68.93	64.77	73.08
3 months	29.29	24.51	34.08	3 months	28.85	24.03	33.68
1 year	22.78	17.81	27.76	1 year	17.24	12.26	22.21
2 years	41.04	30.47	51.61	2 years	34.08	23.58	44.58
Older				Older			
Preoperative	55.54	51.23	59.86	Preoperative	66.34	61.83	70.84
3 months	26.90	22.49	31.31	3 months	32.25	27.67	36.82
1 year	20.65	16.00	25.29	1 year	21.35	16.51	26.18
2 years	36.77	27.60	45.93	2 years	33.64	23.55	43.73
Preoperative pain duration				Preoperative pain duration			
< 1 year				< 1 year			
Preoperative	57.84	54.47	61.22	Preoperative	68.92	65.49	72.35
3 months	27.25	23.61	30.89	3 months	30.61	26.90	34.32
1 year	19.76	15.98	23.55	1 year	18.21	14.37	22.04
2 years	35.40	27.77	43.03	2 years	31.71	23.75	39.66
> = 1 year				> = 1 year			
Preoperative	59.22	52.54	65.89	Preoperative	63.11	56.37	69.85
3 months	31.63	24.44	38.82	3 months	31.89	24.35	39.43
1 year	29.47	21.58	37.35	1 year	24.76	16.53	32.99
2 years	52.72	35.18	70.26	2 years	41.72	23.13	60.30
Preoperative back pain severity				Preoperative back pain severity			
Milder				Milder			
Preoperative	35.78	32.20	39.37	Preoperative	58.02	53.85	62.19
3 months	22.83	18.87	26.78	3 months	25.67	21.14	30.20
1 year	19.02	14.85	23.19	1 year	15.29	10.51	20.08
2 years	24.36	14.76	33.96	2 years	26.89	15.47	38.32
Worse				Worse			
Preoperative	81.11	77.85	84.36	Preoperative	78.22	74.04	82.39
3 months	34.90	30.97	38.84	3 months	36.01	30.86	41.15
1 year	24.33	20.03	28.62	1 year	23.97	18.36	29.58
2 years	49.37	39.77	58.98	2 years	42.10	29.39	54.82
Preoperative leg pain severity				Preoperative leg pain severity			
Milder				Milder			
Preoperative	48.00	43.96	52.04	Preoperative	47.72	43.79	51.65
3 months	26.52	22.09	30.95	3 months	27.46	23.05	31.88
1 year	22.48	17.66	27.30	1 year	21.02	16.23	25.81
2 years	35.87	24.18	47.57	2 years	28.40	16.01	40.78
Worse				Worse			
Preoperative	68.50	64.15	72.84	Preoperative	87.86	84.46	91.25
3 months	29.91	24.77	35.05	3 months	32.52	28.37	36.67
1 year	20.60	15.09	26.11	1 year	16.39	11.96	20.82
2 years	40.56	29.34	51.78	2 years	39.45	30.17	48.74

LR-test showed that the differences in repeated measures were not significantly affected by a slope but only by an intercept – *p*-values > 0.05. Thus, further MIXED analyses were conducted employing intercepts only. For both back pain and leg pain, the change in pain severity over time was statistically significant with *p*-values < 0.001. There were no statistically significant interactions for any of the studied grouping variables, which suggested that the shapes of the trajectories for every grouping variable were similar.

**Fig. 1:**
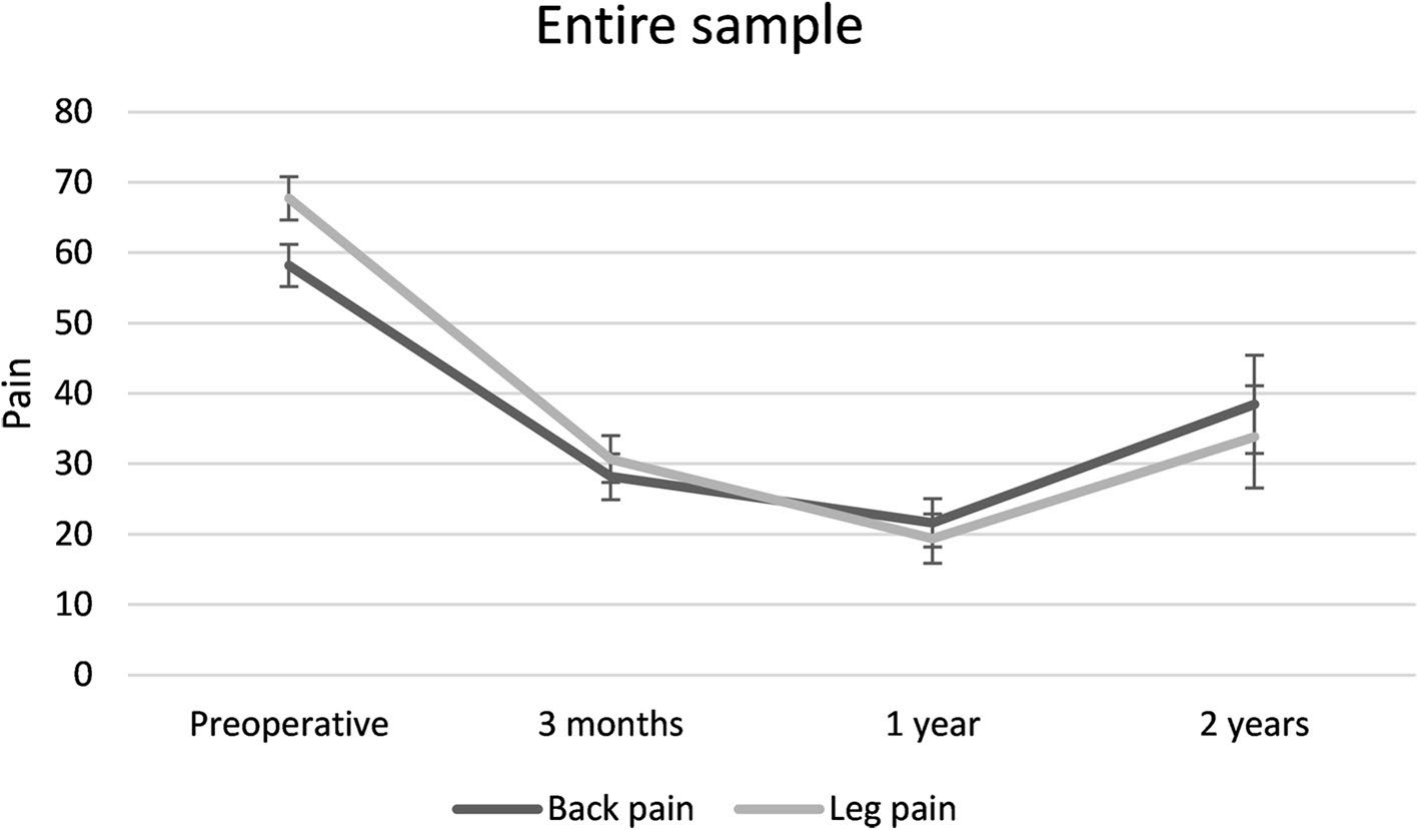
Changes in back and leg pain intensity over time along with 95% confidence interval error bars

As shown in Fig. 1, changes in back and leg pain severity demonstrated similar developmental trajectories. Pain level steeply decreased during the first three months and less steeply till the first year after the surgery. After one year, the pain level was increasing until the end of the two-year follow-up. However, even after that slight increase, pain severity was remained around 30–40/100 points of VAS, which was well below the initial level. Even if the initial pain levels were different, the 95% CIs of pain severity estimates overlapped at each postoperative time point.

**Fig. 2:**
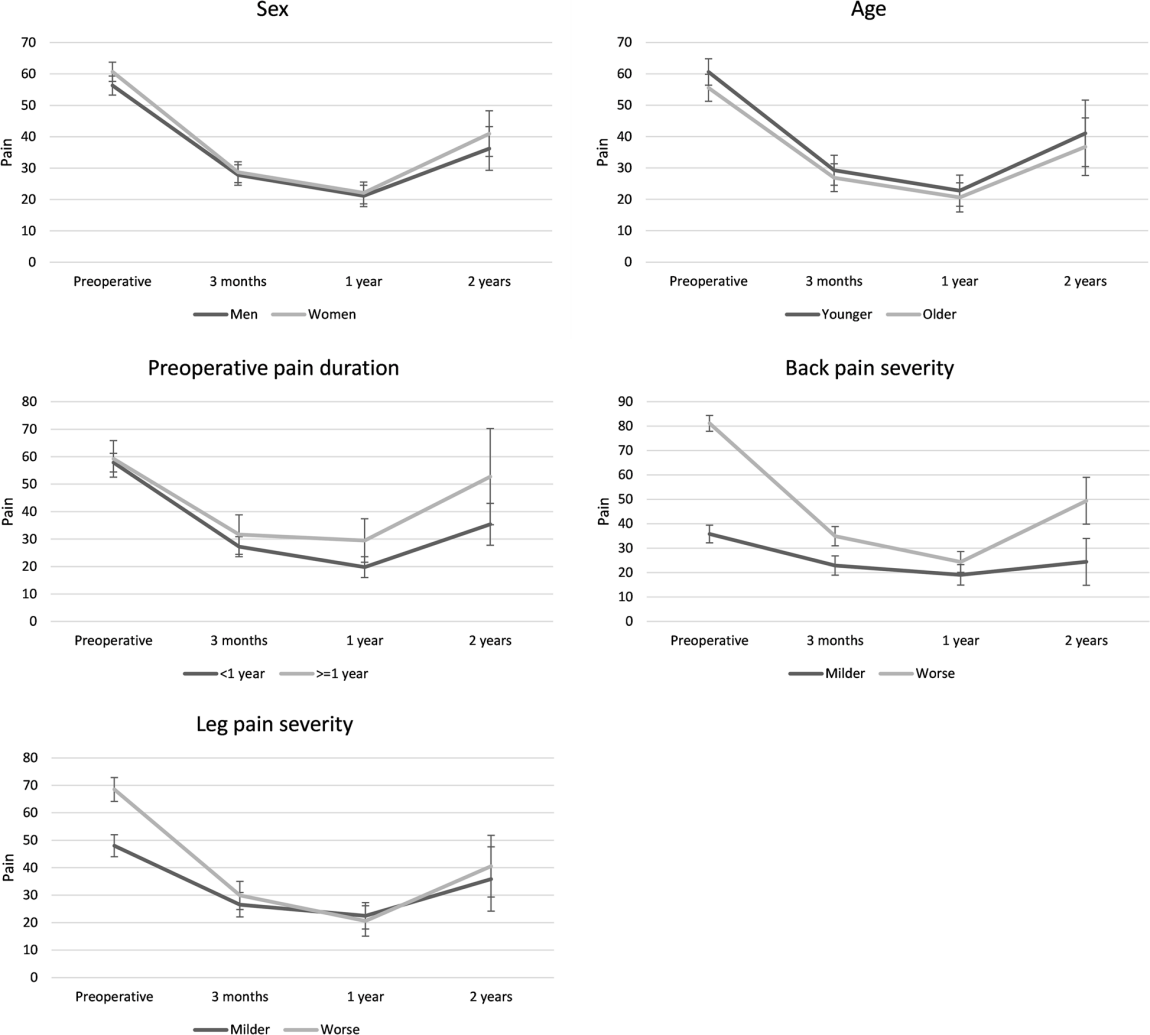
Changes in back pain intensity over time grouped by different variables along with 95% confidence interval error bars

**Fig. 3:**
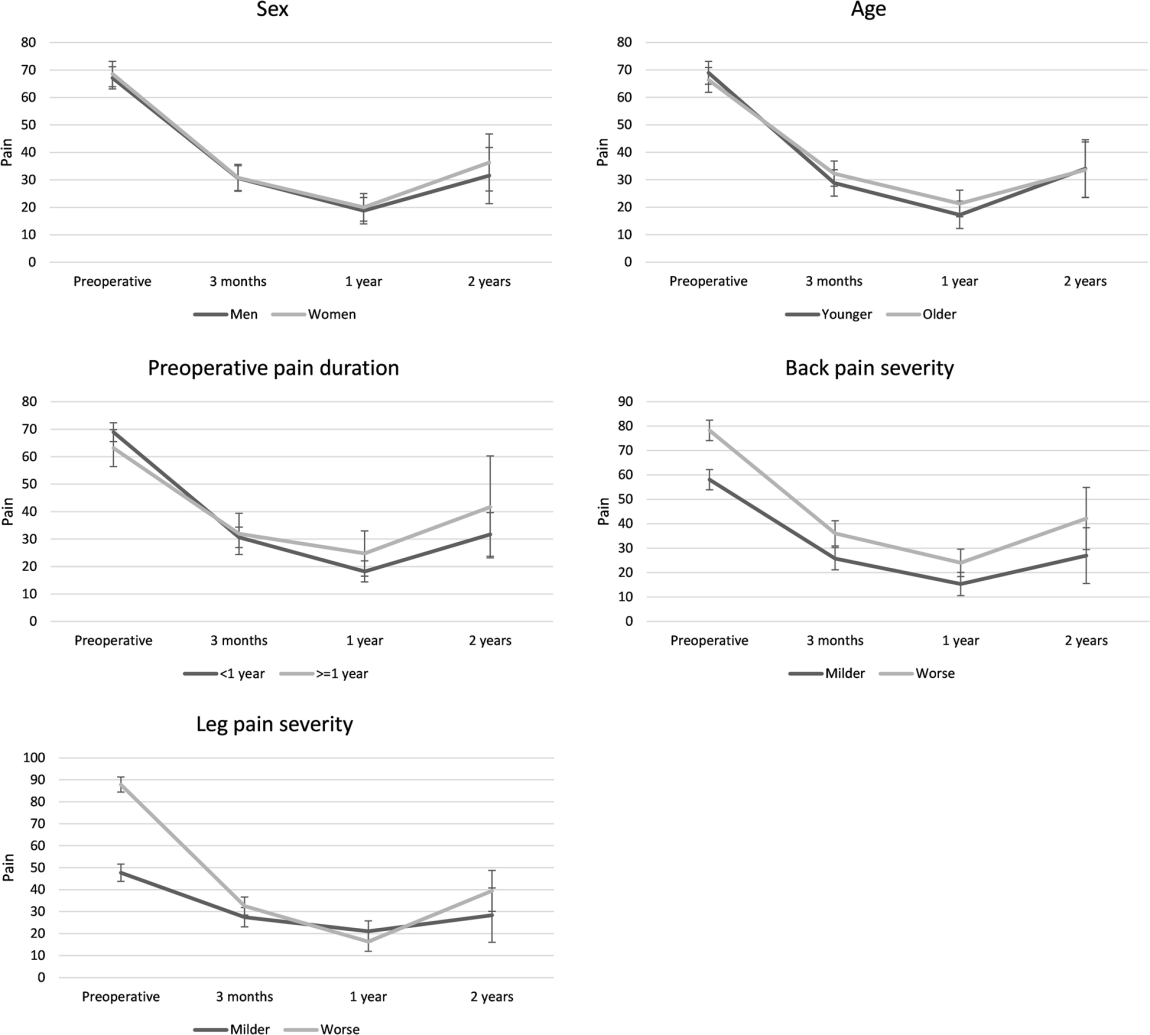
Change in leg pain intensity over time grouped by different variables along with 95% confidence interval error bars

The trajectories of changes in pain levels were similar for both back and leg pain regardless of sex, age or preoperative pain duration (Figs. 2 and 3). The 95% CIs of pain severity estimates overlapped at all four time points.

Regardless of differences in initial leg pain intensity, the trajectories of changes in pain severity were similar for both back and leg pain – the 95% CIs overlapped at each postoperative time point (Figs. 2 and 3). Also, initial differences in back pain intensity were associated with only one significant postoperative difference in pain levels – severe initial pain correlated with slightly more severe pain three months after the surgery as well as at the end of the two-year follow-up (Fig. 2).

## Discussion

This register-based study among 353 patients undergoing lumbar microdiscectomy investigated if a change in back and leg pain severity is associated with sex, age and preoperative pain during two years after the surgery. Back and leg pain demonstrated similar developmental trajectories. Pain level decreased during the first year after the surgery and slightly increased after that. However, at the end of the two-year follow-up, pain level was still well below the initial level. Sex, age or preoperative pain duration were not significantly associated with the differences in studied trajectories. Only the intensity of preoperative back pain was linked to significant differences in pain level during the postoperative repeated measures – people with more severe preoperative back pain reported worse pain three months and two years after the surgery.

The generalizability of the findings might be limited due to several issues. Only a few demographic variables were available for the analysis. Thus, it is possible that there might be several important factors, other than age, sex or preoperative pain, which might affect the changes in pain severity after the surgery. For example, an educational level, an occupation, the content of rehabilitation arranged before or after the surgery, or preoperative psychological traits like fear or anxiety might affect the results. However, two out of four main factors (preoperative pain, anxiety, age, and type of surgery) related to alleviating pain, according to a previous review on postoperative pain, were available [19]. The study was set at a university surgery clinic, which is a highly specialized unit, and therefore, the results might be different in lower-level units. Unfortunately, a longer follow-up was not possible to arrange. The register was part of an electronic patient record. Due to the hospital policy, in such situation, patients could be contacted only when it is necessary concerning their treatment and not for a research purpose. Indeed, a follow-up extended to five or 10 years after surgery could provide important additional information, especially considering the fact that some of the curves were pointing upwards at the end of two-year follow up. The results might also be affected by a regression towards the mean, contextual effects or placebo effects. While the dichotomization of independent variables may improve the interpretability of the results, it leads to a loss of study power.

Surgery due to intervertebral disc displacement focuses primarily on relieving sciatic leg pain [9]. There is uncertainty on how much microdiscectomy affects back pain [10]. Only a few previous studies have suggested that also back pain may be substantially relieved by the procedure in question [8]. This is in line with the present results, which did not only suggest simultaneous improvement in both back and leg pain, but observed essentially the same magnitude of these changes. While the severity of leg pain was a little worse than back pain at the baseline, they both decreased to a similar level in two-year follow-up.

Several speculations could be introduced to explain the similarity between postoperative changes in back and leg pain. Additionally to mechanical compression, intervertebral disc displacement causes local inflammation. When burst out prolapse mass is dissolved by surgery or by natural course over time, inflammation starts to ease, which affects both local and radicular pain. Also, patients with intervertebral disc displacement may often consider pain in buttocks as “back pain” and not as “radicular pain”, which it in fact is. This may affect the interpretation of surgery outcome when attempting to distinguish local back pain and radicular leg pain. Both local and irradiating pain may affect the mechanics of low back structures. It might be difficult to achieve a painless position and e.g., sitting is often more painful than standing. When radicular pain is gone, also low back mechanics are normalized.

Certainly, controls would be required in order to draw strong conclusions concerning the effect of surgery on pain severity. Nevertheless, these results are encouraging – most of the patients experienced pain relief in a relatively long run after the surgery.

In line with previous research, no significant effect of sex or age on the magnitude of postoperative pain relief was found [7, 13, 15]. A few previous studies from the same research team have suggested that older age and female sex might predict worse outcome of microdiscectomy [12, 15, 20].

Based on common disputes on the “right timing” for discectomy, a correlation between prolonged preoperative pain and a better surgery outcome could be expected [17]. It could be assumed that pain is not completely related to a present prolapse situation, but, instead, partially caused by other reasons, e.g., degenerative spinal changes. These additional reasons may not be cured by a discectomy and pain may remain. Also, prolonged pain may cause psychological or social effects on the functioning of patients. Due to this effect, pain may continue even if the morphological cause is cured. However, there was not such an association observed in this study. Instead, only the preoperative level of back pain was significantly related to better pain relief after surgery.

Only pre-operative back pain severity was associated with differences in the shapes of developmental trajectories, which were otherwise alike, suggesting that this association reflects a mean change in pain severity over time that is dependent on the baseline score. A significant association between baseline and pain developmental trajectories may potentially be explained by contextual factors, which have been left outside the scope of this study. Such factors might be related to differences in psycho-social situations (which exacerbated pain or the other way around) before surgery, or to a possibility that worse pain was associated with more severe concomitant diseases, other than local disorders in the lumbar spine.

The present results should be confirmed by additional research conducted in different settings, diverse populations and wider sets of available independent variables. Especially, a randomized controlled trial may be of great interest to compare the effect of surgery with the effect of placebo surgery on pain development. However, arranging such a trial could be problematic due to ethical issues. While this study demonstrated the average changes in pain severity after surgery, a group-based trajectory analysis on the topic would be of interest. It is possible, and even probable, that some groups of patients may demonstrate trajectories, which substantially differ from the average ones. Identifying such groups may be of great interest especially concerning the selection of patients, who may benefit from the surgery the most.

## Conclusions

After microsurgical discectomy, developmental curves for both back and radicular leg pain demonstrated similar patterns. Pain intensity decreased during the first year after the surgery and slightly increased after that remaining, however, below the preoperative level. Age, sex, preoperative pain duration or preoperative intensity of leg pain were not associated with significant differences in the trajectories of pain severity after surgery. In this study, severe preoperative back pain was the only factor, which was significantly associated with worse postoperative trajectory of pain intensity.

## Data Availability

The datasets used and/or analysed during the current study are available from the corresponding author on reasonable request.
